# Editorial: Acknowledging Our Work as Reviewers

**DOI:** 10.1523/ENEURO.0031-17.2017

**Published:** 2017-01-31

**Authors:** Christophe Bernard

eNeuro is pleased to announce that now, in addition to your publications, you will be able to build a history of the reviews you performed. As mentioned in a previous editorial, reviewers play an integral role in the progression of the collective work that is Science. Reviewers help authors build stronger studies. If our work as scientists can be accessed via our publications, our work as reviewers is not quantifiable yet. Now, once you have a performed a review for eNeuro, you will have the opportunity to record your review in your ORCID profile. You can choose whether you want this information to be public or not.

As a reminder, ORCID is a non-profit community-based effort to give authors persistent digital identifiers, in other words, each researcher gets a unique number. Through ORCID, a profile is created that includes all of your publications, grant submissions, and more. Registering with ORCID offers multiple advantages, such as faster form filling when submitting a paper, evaluation of one’s work by institutions, etc. Another major advantage is to simplify the method for connecting to the collection of your body of work throughout your career, while other things can potentially change, such as your name, location, institution, and even discipline.

No doubt that most, if not all, journals will adopt the same procedure. That way, there will be a quantifiable way to assess your work as a reviewer. It can become an important piece of information in your CV.

